# Sex Disparity in Cancer: Role of Autophagy and Estrogen Receptors

**DOI:** 10.3390/cells14040273

**Published:** 2025-02-13

**Authors:** Rosa Vona, Camilla Cittadini, Elena Ortona, Paola Matarrese

**Affiliations:** Center for Gender-Specific Medicine, National Institute of Health, 00161 Rome, Italy; camilla.cittadini@iss.it (C.C.); elena.ortona@iss.it (E.O.)

**Keywords:** autophagy, estrogen receptor, gender disparity

## Abstract

Autophagy, a cellular process essential for maintaining homeostasis, plays a fundamental role in recycling damaged components and in adapting to stress. The dysregulation of autophagy is implicated in numerous human diseases, including cancer, where it exhibits a dual role as both a suppressor and a promoter, depending on the context and the stage of tumor development. The significant sex differences observed in autophagic processes are determined by biological factors, such as genetic makeup and sex hormones. Estrogens, through their interaction with specific receptors, modulate autophagy and influence tumor progression, therapy resistance, and the immune response to tumors. In females, the escape from X inactivation and estrogen signaling may be responsible for the advantages, in terms of lower incidence and longer survival, observed in oncology. Women often show better responses to traditional chemotherapy, while men respond better to immunotherapy. The action of sex hormones on the immune system could contribute to these differences. However, women experience more severe adverse reactions to anticancer drugs. The estrogen/autophagy crosstalk—involved in multiple aspects of the tumor, i.e., development, progression and the response to therapy—deserves an in-depth study, as it could highlight sex-specific mechanisms useful for designing innovative and gender-tailored treatments from the perspective of precision medicine.

## 1. Introduction

Maintaining cellular homeostasis is essential for the survival of all organisms. Eukaryotes, from simple yeasts to the most complex mammals, have developed a genetically driven mechanism that regulates the degradation of damaged or obsolete cellular components and organelles while ensuring the recycling of the basic components from which cells are able to initiate the synthesis of new molecules and organelles [1]. This process, known as macroautophagy or autophagy, involves the sequestration of cytoplasmic compartments, containing molecules and/or organelles to be recycled, inside a double-membrane phagophore, whose destiny is to fuse with the lysosome within which proteases ensure the degradation of the various cellular components [2,3,4].

Autophagy plays a fundamental physiological role; therefore, its dysregulation is implicated in all human diseases, from neurodegenerative to cardiovascular and to neoplastic pathologies [5,6].

It is now clear that all human diseases, either infectious or noninfectious, show sex differences in one or more aspects, such as incidence, clinical manifestations, progression, prognosis and the response to therapy [7,8]. Tumors, even those that do not strictly involve the reproductive organs, which are considered sex-specific, are no exception [9]. In humans, it has been widely confirmed that cancer shows a higher incidence and higher mortality in males [9]. Multiple and complex factors determine this bias—some sex-related, others gender-related. It is in fact particularly important to distinguish between sex (as a biological variable) and gender (as a social variable), both for epidemiological and for biomedical research [10]. Among the biological variables, hereditary genetics, epigenetics and sexual hormones play a complex and intricate role that makes it difficult to investigate the relative weight of each one and is presumably different for each clinical case [11].

Epigenetics is strongly influenced by external factors, such as infections, food, traumatic experiences, chemical pollutants and physical factors (for example, heat and cold), and it can be transmitted to next generations. In females, even the inactivation of one of the two X chromosomes, which are random and incomplete (about 15% escape inactivation), strongly affects the expression of both the genes and non-coding RNAs (long non-coding RNAs, circular RNAs and microRNAs) present in the areas that escape inactivation, which can have an impact on the onset and progression of cancer [12]. Sex hormones, in turn, through interactions with specific receptors, ubiquitously expressed in all tissues in both males and females, can regulate the gene expression, epigenome and metabolome of tumors. On the other hand, the downstream signaling network of estrogen has also been shown to be implicated in the regulation of autophagy, differently depending on the specific receptors involved.

It therefore appears essential to examine the potential crosstalk between estrogen signaling and autophagy to better understand the mechanisms underlying the observed sex differences in tumors and to design innovative, more effective and personalized therapeutic approaches.

## 2. Autophagy

Autophagy, a term of Greek origin, describes the lysosomal degradation (phagy) of part of the cell itself (auto). A few different forms of autophagy have been described, but here, we will be discussing macroautophagy (also referred to as autophagy). This process occurs in all cells and provides a mechanism for maintaining cytoplasmic turnover and removing damaged organelles and protein aggregates. Autophagy is also upregulated in response to physiological conditions, such as nutrient deprivation, and various pathological stresses, including hypoxia, infection and many others, allowing cells to adapt to environmental and developmental changes [2,13]. In addition to its traditional degradative functions, autophagy has been shown to be involved in the secretory pathways of both yeast and mammalian cells [14].

### 2.1. Biogenesis of the Autophagosome

**Figure 1:**
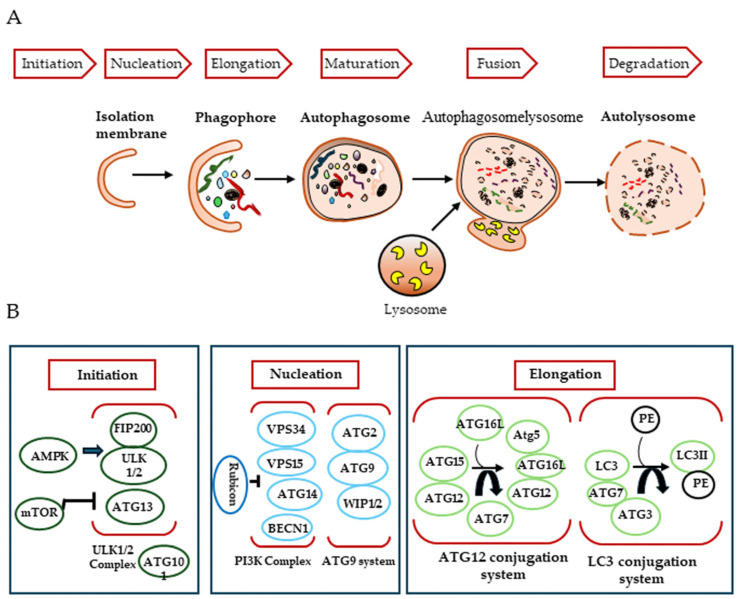
Mechanisms of autophagy induction. (**A**) During macroautophagy, dynamic membranes form phagophores and cytosolic components are sealed into autophagosomes that fuse with lysosomes to degrade cytosolic components, leading to autolysosome. (**B**) The major complex involved in the formation of autophagosomes.

In mammals, the autophagic process consists of three steps, initiation, nucleation and elongation, which begin with the genesis of the autophagosome (Figure 1). Autophagosomes are thought to originate at various membrane sites in the cytoplasm. Proteins that mediate the autophagic process are known as ATG (autophagy-related) proteins.

The first step of this mechanism involves the formation of a phagophore, a cup-shaped structure that will later develop into the autophagosome, and is mediated by the ULK1/2-FIP200-ATG101 complex. This complex is negatively regulated by mTORC1 (mammalian target of rapamycin complex 1), which associates with it when nutrients, growth factors, amino acids and glucose signals are abundant. Conversely, in their absence or under starvation conditions, mTORC1 dissociates, leading to activation of the complex to induce autophagy [15,16].

Nucleation requires the activity of PI3K (the class III phosphatidylinositol 3-kinase) complex, which generates the phosphatidylinositol (3)-phosphate required to recruit other autophagy-related proteins to the phagophore assembly site. This process is mediated by a complex composed of human vacuolar protein sorting associated protein 34 (hVPS34, a lipid kinase), Beclin 1 (BECN1) and p150, in addition to several other Beclin 1 binding proteins [17,18]. At this stage, several key modulators interact with the class III PI3K complex to regulate autophagy. ATG14-like protein (ATG14L, also called Barkor) is thought to enhance autophagy by targeting the class III PI3K complex at sites of autophagic biogenesis via its interaction with BECN1. Another protein, UVRAG, is also known to promote autophagy by interacting with Beclin 1 [19]. The UVRAG-associated class III PI3K complex also provides a platform for the binding of Bif-1, which is involved in phagophore membrane curvature [20], and Rubicon, which negatively regulates autophagosome maturation [21]. Other proteins that interact with Beclin 1 have been identified: Ambra 1, which promotes autophagy [22], and the apoptosis regulator Bcl-2 [23] and the serine/threonine protein kinase AKT [24], which have inhibitory effects.

The final step, autophagosomal membrane elongation, is controlled by two ubiquitin-like protein conjugation systems: ATG12-Atg5 and ATG8/LC3 [25]. How autophagosomes mature and close after biogenesis is poorly understood. ATG2A and ATG2B, the soluble N-ethylmaleimide-sensitive attachment protein receptor (SNARE), the small GTPase RAB7 and the class C VPS complex are required to close the phagophore and form the autophagosome. Finally, the autophagosome fuses with the endosome–lysosome to form the autolysosome, which is degraded along with its luminal contents [26]. Autophagosomes can acquire their membrane from a variety of sources, including the endoplasmic reticulum, the outer mitochondrial membrane, the plasma membrane and the Golgi apparatus. In addition, ATG8/LC3 recruits adaptor proteins, such as p62 and NBR1, to autophagosomes to mediate the selective autophagy of various cellular structures, protein aggregates and microorganisms [27]. Autophagosome maturation is uniquely directed by its movement along the cytoskeletal networks of microfilaments and microtubules [28].

### 2.2. Key Regulators of the Autophagic Process

Metabolic stresses involving nutrient and/or growth factor deprivation are potent inducers of autophagy; they act through specific sensors such as mTORC1/2 and adenosine monophosphate 5′-activated protein kinase (AMPK). In particular, mTORC1 is the best characterized modulator of autophagy and is a central sensor of the metabolic status in the cell. It inhibits autophagy by directly phosphorylating the ULK1 complex and induces autophagy when inhibited by rapamycin. A variety of anabolic inputs, including cellular energy status and the presence of amino acids and growth factors, stimulate the activity of mTORC1. Conversely, mTORC1 is inhibited when amino acids are scarce, when growth factor signaling is reduced and/or when ATP levels fall, leading to the activation of the autophagy process [18,29].

Glucose restriction and energy restriction can also activate AMPK, a positive regulator of autophagy. In fact, low-energy states promote, through AMPK, the inhibition of mTORC1. Furthermore, AMPK can regulate the activity of the class III PI3K complex, which directly phosphorylates some proteins, including Raptor, a member of mTORC1, ULK1 and BECN1, thus activating autophagy [29]. Pharmacological activators and inhibitors of autophagy, such as BH3 mimetics and spautin, respectively, can affect the activity of the class III PI3K complex. Lysosomal protease inhibitors (e.g., cystatin B) and lysosomotropic agents that increase lysosomal pH (e.g., chloroquine, hydroxychloroquine, bafilomycin A) also inhibit degradative activity by acting on the terminal stages of the autophagic process [30].

Akt signaling and the transcription factor EB (TFEB) can modulate the transcriptional regulation of some autophagy-related and lysosomal genes in response to nutrient starvation [31]. In low oxygen conditions, the transcriptional regulator hypoxia-inducible factor 1 (HIF-1) has been shown to promote autophagy [32].

P53 can activate autophagy in response to a variety of DNA damage inducers, including chemotherapeutic agents, UV radiation and ionizing radiation, by transcriptionally upregulating several genes encoding lysosomal proteins and nuclear *ATGs* [13,33,34].

## 3. Autophagy and Cancer

A plethora of diseases, such as metabolic, neurodegenerative, infectious and inflammatory diseases, autoimmune disorders, and cancer, as well as aging, are associated with alterations in the autophagic process [35]. Cancer was one of the first diseases linked to autophagy dysfunction. It has been proposed that BECN1 acts as a tumor suppressor [36], and more recently, low levels of BECN1 have been linked to a worse prognosis in gastric, colorectal and pancreatic, as well as breast, cancer. By contrast, high levels of its expression were associated with improved survival in high-grade gliomas, hepatocellular carcinoma and B-cell lymphomas [37].

Experimental data have shown that autophagy can actually be both a suppressor and a promoter of cancer. This dichotomous effect may depend on both the context and the stage of tumor development [38]. For example, autophagy could promote the survival of damaged cells that could give rise to cancer. In the same vein, elevated levels of autophagy could be responsible for the resistance of tumor cells to drug treatments so that the inhibition of autophagy could represent a strategy to reduce drug resistance and, therefore, improve therapeutic effects. It has also been observed in several experimental settings that autophagy may play an important role in tamoxifen resistance in breast cancer [39,40].

On the other hand, other studies suggest that increased autophagic death may have antitumor effects, especially in tumors that are poorly susceptible to apoptosis. In addition, autophagic processes decisively influence the antitumor immune response [41,42].

As mentioned above, autophagy can play a tumor suppressor role, especially during the transformation phase, limiting inflammation and the consequent tissue damage, and genome instability, known to promote carcinogenesis. The elimination of damaged mitochondria through mitophagy reduces the formation of ROS, which, by inducing oxidative stress, would favor DNA damage and, consequently, genomic instability. This series of events would trigger an inflammatory response with the production of cytokines, capable of further promoting cancer growth by creating a favorable microenvironment. Furthermore, the removal of malfunctioning mitochondria by autophagy preserves cellular bioenergetic functions, whose alteration is a fundamental step in tumor transformation and progression. Defective autophagy leads to the accumulation of non-functional proteins and organelles that generate oxidative stress with a consequent genotoxic effect, thus triggering an evil loop that promotes tumor progression [43]. Autophagy may, therefore, play its protective role through multiple mechanisms: maintaining energy homeostasis, reducing oxidative stress and eliminating damaged proteins and organelles.

In accordance with this, an accumulation of p62/SQSTM1 has been observed in many human tumors, which could represent not only a biomarker but also evidence of an altered autophagy in tumor cells [44]. Moreover, increased p62 and nuclear factor erythroid 2-related factor 2 (NRF2), a transcription factor that mediates the cellular antioxidant response under conditions of oxidative stress, has been reported in a subset of NSCLCs with poor prognosis [45,46].

Alterations in BECN1 and some *ATG* genes, such as *ATG5* and *ATG4*, which limit the efficacy of autophagic processes, have also been observed in liver, breast, prostate and ovarian tumors, confirming that autophagy plays a tumor-suppressive role in different tissue contexts [47,48,49].

Autophagy also plays an indirect role in tumor suppression through its effects on the immune system and in many aspects of immunosurveillance. Indeed, it is involved in the development of adaptive immunity, in the elimination of pathogens by innate immunity and in the secretion of cytokines (secretory autophagy) and other immune signaling molecules [43]. Autophagy contributes to tumor suppression also through the maintenance of controlled levels of inflammation, which is one of the components of the microenvironment that promotes tumor growth and progression. Autophagy limits inflammation by regulating the inflammasome, a protein complex involved in the activation and release of inflammatory cytokines in response to pathogen- and xenobiotic-induced damage. This regulation occurs at two levels: degradation of the inflammasome and clearance of inflammasome activators [50].

On the other hand, it has also been shown that in the later stages of cancer development, autophagy can promote the survival of tumor cells. Indeed, it is thought that autophagy could represent a pro-cancer mechanism, as it would allow cell survival in stressful conditions associated with transformation events, metastasis and anticancer treatments [51]. Autophagy contributes to cancer development and progression also by maintaining cancer cell metabolism by contributing to the recycling of metabolic substrates.

Autophagy-supported metabolic adaptation is one of the key requirements for cancer to develop and progress [49,52]. The importance of autophagy in supporting cancer metabolism relates to its role in facilitating glycolysis [53]. Indeed, the Warburg effect, a switch from oxidative phosphorylation to aerobic glycolysis to produce ATP from glucose, has been observed in some tumors. In this way, tumor cells may be able to direct glycolytic metabolites to biosynthetic pathways. Furthermore, the importance of the autophagic process is evident in the maintenance of mitochondrial homeostasis. By eliminating both structurally and functionally defective mitochondria, autophagy may counteract the depletion of tricarboxylic acid (TCA) cycle intermediates and the impairment of mitochondrial respiration [54].

Recent studies have highlighted the complex relationship between autophagy and lipid homeostasis [55,56]. In fact, autophagy is essential for the degradation of intracellular lipids by facilitating the basal turnover of lipids stored in lipid droplets [55], and it also helps repair damaged cellular structures and provides energy for anabolic processes that require lipid components. In the case of a short-term need for lipids or starvation, autophagy is enhanced to make lipids stored in fatty acids available. In contrast, the inhibition of autophagy prevents lipid degradation, resulting in the accumulation of cellular lipids. Such lipid accumulation suppresses autophagic mechanisms by promoting further lipid storage and larger lipid droplets [57].

Sphingolipid metabolism has been found to be dysregulated in cancer, and sphingolipids have been shown to play a critical role in regulating the growth and death of tumor cells [58]. In particular, sphingosine-1-phosphate (S1P) is a cell-fate-determining molecule that facilitates the development of gastrointestinal tumors by activating inflammatory signals [59].

Estrogens, through the activation of sphingosine kinase 1 (SphK1), have also been observed to increase the intracellular concentration of S1P, proliferation and cell survival, at least in breast cancer [60].

An abnormal metabolism of S1P has also been observed in lymphangiomyomatosis, a devastating pulmonary disease that affects only women, characterized, from a biochemical point of view, by the chronic activation of mTORC1 and consequent inhibition of autophagy. Interestingly, it was observed that inhibiting S1P synthesis and simultaneously activating mTORC1 by rapamycin induced the autophagic death of tumor cells [61].

Based on the above, the pharmacological modulation of the enzymes or metabolites involved in lipid metabolism may be potentially of interest in cancer therapy [62].

Autophagy is therefore thought to act as a stress response mechanism, facilitating tumor cell survival under conditions of nutrient deprivation [63], hypoxia [64], endoplasmic reticulum stress [65], anticancer therapy [66] and the loss of contact with the extracellular matrix during invasion and metastasis, thereby counteracting anoikis [67].

It is now known that tumor progression does not only depend on the proliferation of cancer cells but also, and above all, on the way in which these cells interact with other actors present in the tumor microenvironment, such as fibroblasts, immune cells, pericytes and endothelial cells [68]. These interactions are thought to create a favorable environment, contributing to tumor progression by promoting angiogenesis, remodeling the extracellular matrix, facilitating escape from immune surveillance and secreting growth factors, or they can create an unfavorable environment for the tumor, contributing to healing and the response to therapy [69]. In this context, autophagy can mediate critical metabolic changes in cancer-associated fibroblasts (CAFs) that promote tumor progression through the metabolic coupling of stromal cells and cancer cells [70]. In addition, autophagy may be a modulator of tumor cell interactions with the immune system. It facilitates the escape of cancer cells from immune cell-mediated clearance and creates a pro-tumorigenic environment [71].

## 4. Sex Differences in Autophagy

Sex differences have been observed in both physiological and pathological autophagy. These differences are determined both by sex steroid hormones (androgens and estrogens) and genetically by the unique chromosomal configuration of the two sexes (XX versus XY). In vitro studies performed on cells isolated from male or female tissues highlighted a greater propensity of XX cells for autophagy compared to XY cells. Thanks to this, it has been hypothesized that female cells could survive better than male cells in unfavorable environmental conditions. Accordingly, female cells have been observed to have lower basal levels of autophagy than male cells but a greater capacity to modulate it following stress [72,73].

Some genes involved in autophagy (*ATG*) are located on the X chromosome [74]. For example, *ATP6AP2* (ATPase H+ transport accessory protein 2) and *LAMP2* (lysosome-associated membrane protein 2, a lysosomal protein involved in the fusion between autophagosomes and lysosomes) are located on the X chromosome, and their mutations are associated with pathological conditions in humans [75]. The same applies to some proteins of the RAB family, GTPases that regulate vesicular trafficking and are directly involved in autophagy, such as RAB9A, RAB9B, RAB33A and RAB39B. RAB33A together with ATG16 L1 participate in the genesis of the autophagosome, and RAB39B is involved together with ULK1 in the initiation of the autophagic process, whereas RAB9A and RAB9B play a role in mitophagy [76,77,78].

These are just a few examples of genes involved in autophagy located on the X chromosome that may contribute to the observed sex differences. In this review, we will focus on the role of estrogen receptors (ERs) in autophagic processes and, through them, on the role played by these hormones in the development and progression of some human tumors.

## 5. Estrogen Receptors and Cancer

Cancer is the second leading cause of death worldwide. Incidence and mortality rates differ significantly by gender, being higher in men than in women. In addition, for some tumors, gender differences have also been observed in prognosis, the response to therapy, and the adverse effects of therapy. An important study has shown that the tumor suppressor genes present in the areas of the X chromosome that escape inactivation may play a role in the female advantage observed in some tumor types [79], such as hepatocellular carcinoma, where the male-to-female ratio is, on average, between 2:1 and 4:1 [80].

However, a growing body of evidence suggests that for many non-sex-specific tumors, the dysregulation of ER signaling pathways may contribute to the observed sexual dimorphism. Such a hormonal contribution was recognized until a few years ago only for sex-specific tumors, that is, tumors involving the male and female reproductive systems (e.g., breast, uterus, ovary, prostate), obviously sensitive to sex hormones [81,82].

**Figure 2:**
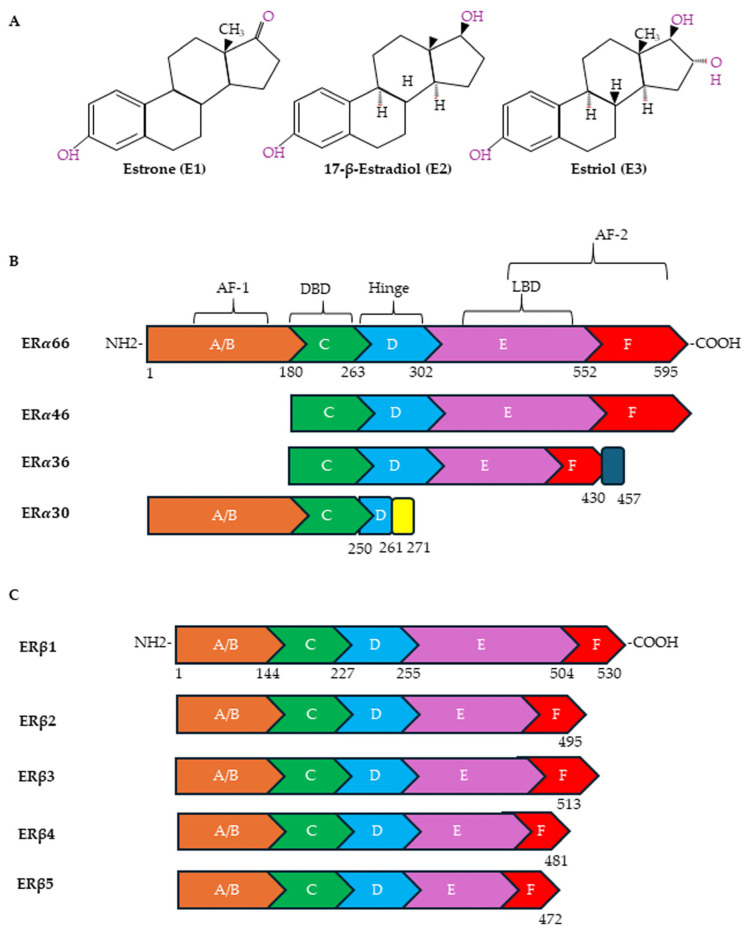
Chemical structures of estrogens and ER domains. (**A**) Human estrogens are estrone (E1), 17-β-estradiol (E2) and estriol (E3). Structure of canonical forms and different variants of ERα (**B**) and ERβ (**C**). Full-length ERα contains 595 amino acids, whereas ERβ contains 530 amino acids. Both ERα and ERβ have six distinct regions (A–F). A ligand-independent transcriptional activation function (AF-1) domain is located in the NH2-terminal A/B region. The major functional difference between ERα and ERβ is due to the AF-1 domain. The E and F regions also contain a ligand-dependent transcriptional activation domain (AF-2), which is involved in activating transcription in response to estrogen. Both AFs contain protein–protein interaction regions. The DNA-binding domain (DBD) (region C) is required for the dimerization of the receptor and for the sequence-specific binding of ERs to DNA sequences in target genes designated as EREs. Through acetylation or ubiquitination, the hinge domain (region D) is responsible for nuclear localization and ER activity. The ligand-binding domain (LBD) is in the region that plays a role in the binding of ligands and the dimerization of ERs.

Estrogens exert their effects by binding to ERs (Figure 2A). The following receptors for estrogens have been identified: ERα and Erβ, located in the nucleus and cytoplasm, and G protein-coupled estrogen receptor-1 (GPER), located on the plasma membrane. ERα and ERβ have also been observed on plasma membranes and on the membranes of organelles such as mitochondria [83,84]. In the plasma membrane, ERs are localized in specific functional domains, the lipid rafts, in which different receptors and signaling proteins are integrated [85]. ERα and ERβ, although sharing some sequence homology, are encoded by two different genes, *ESR1* and *ESR2*, located on different chromosomes (locus 6q25.1 and locus 14q23-24.1, respectively). Both are transcription factors of the nuclear receptor superfamily that regulates the expression of genes involved in cell survival, proliferation, differentiation and reproduction [86]. ERα and ERβ have several alternatively spliced mRNA variants (Figure 2B). In particular, ERα contains, in addition to the full-length ERα-66 protein, shorter variants known as ERα-46, ERα-36 and ERα-30 [87,88]. ERα-46 is present in several types of human breast malignancies, occasionally even exceeding the abundance of ERα-66 protein; ERα-36 can antagonize the action of ERα-66 [89]; and ERα-30 seems to inhibit the expression of ERα-66, acting as a negative regulator [90]. As far as the ERα-36 splice variant is concerned, the literature data would seem to indicate, although not unequivocally, that it plays an important role in tumor progression and in the development of resistance to cancer treatments [88]. ERβ, on the other hand, exists in six different isoforms, ERβ1-6, of which ERβ1 is the full-length isoform (Figure 2C). While ERβ1 is predominantly an oncorepressor, ERβ2, ERβ4 and ERβ5 have been shown to promote breast cancer progression. There is limited data on the role of ERβ3 and ERβ6 [91].

**Figure 3:**
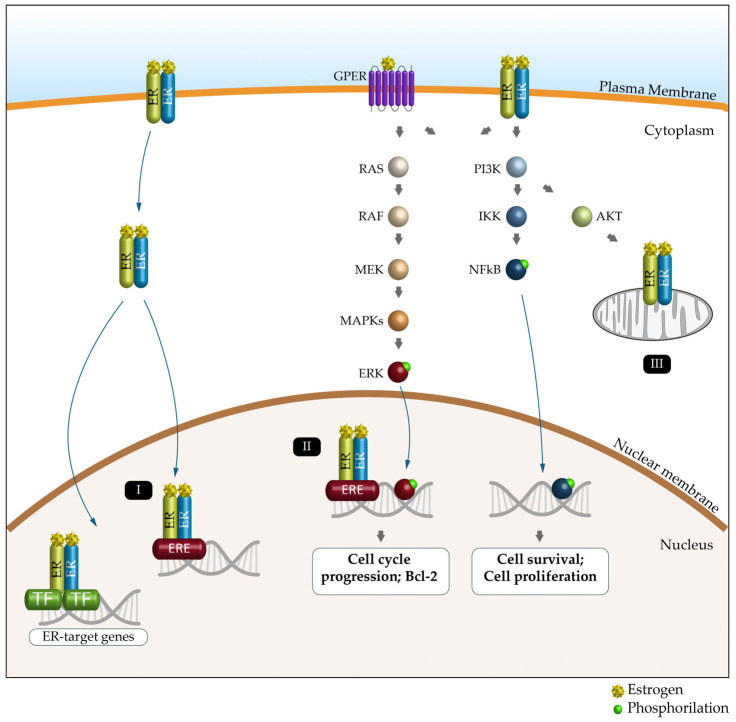
Schematic representation of the ER signaling pathway and its key functions. Estrogen (E) can bind to the estrogen receptor (ERα, ERβ and GPER) to affect the expression and activity of several signaling pathways. Genomic pathway: (I) The E2/ER complex can bind to estrogen response elements (EREs) within the target gene promoter or regulate gene transcription by interacting with other transcription factors (TFs), such as AP-1 and Sp1. (II) The E2/ER complex also activates signaling pathways that phosphorylate (green dot) ERs or other associated transcription factors (e.g., ERK and NFkB) and modulate gene expression. (III) E2-induced cellular and mitochondrial ER/GPER genomic and non-genomic effects regulate mitochondrial respiration, ATP production and ROS formation.

Nuclear ERα and ERβ bind DNA directly through estrogen response elements (EREs) or indirectly through ERE-independent transcription factors such as the nuclear factor kappa-light-chain-enhancer of activated B cells (NF-κB), activator protein 1 (AP1), SP1 and C/EBPβ to induce or repress gene expression [86,92]. ERs located in the plasma or intracytoplasmic membranes produce rapid (non-genomic) signaling effects by modulating intracellular calcium, cAMP and potassium currents, activating phospholipase C and stimulating the PI3K/AKT and ERK pathways (Figure 3) [93]. Estrogen signaling, through its receptors (particularly ERα and ERβ), mediates metabolic and mitochondrial homeostasis, autophagy and epigenetic modifications both in men and in women [94].

Although a role in tumors has also been described for GPER, this review will only focus on classic ERs.

## 6. Autophagy and Estrogen Receptors

Many non-reproductive tumors in men and women show sex differences in incidence, clinical manifestations, prognosis and the response to therapy. Some of these sex differences appear to be related to the autophagic process [75].

In the regulation of sex-dependent autophagy, one of the key elements is represented by sex steroid hormones and their receptors. Androgens and estrogens impact autophagy by regulating the expression of several *ATG* genes. In particular, they influence phagophore genesis and growth by acting on the expression of ATG3, ATG4b, ATG5, ATG7, LC3b and Sqstm1/p62 [74] as well as autophagosome maturation by acting on LC3-II levels [95]. They can also control the final stages of autophagy by regulating the expression of lysosomal proteinases such as cathepsin D. Therefore, steroid hormones can interfere with the autophagic mechanism by modulating all its phases, from induction to degradation [96].

Important evidence for the role of estrogens in autophagy comes from gonadectomized animals; no alteration in MTOR activation was observed following ischemic damage in males, while in females, an increase in MTOR activation was observed, thus suggesting a different role of estrogens and androgens [97].

**Figure 4:**
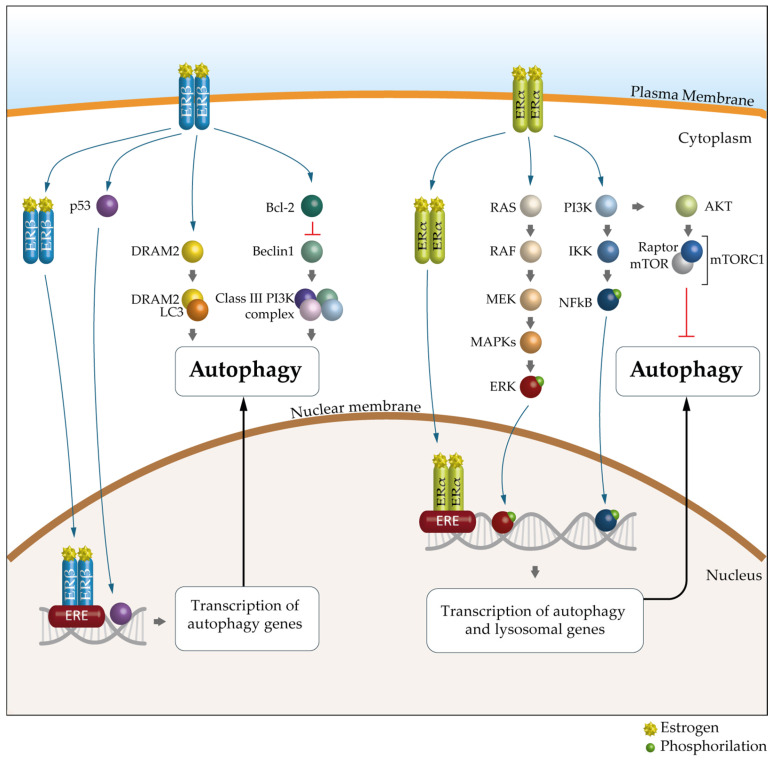
The molecular mechanism of ER-mediated autophagy. The mechanism of ERα-mediated autophagy. Estrogen via ERα could inhibit autophagy through PI3K/AKT/mTOR signaling pathways. mERα appears to promote autophagy mainly through the MEK/ERK signaling axis, through NFkB or through the E_2_/ER complex, which can bind to estrogen response elements (EREs). The mechanism of ERβ-mediated autophagy. ERβ can activate autophagy by binding to the p53 promoter, through classic PTEN/PI3K/AKT/mTOR signaling pathways or through the E2/ER complex, which can bind to EREs. In addition, DRAM2, a key autophagic regulator, is involved in ERβ-mediated autophagy. Furthermore, the presence of ERβ causes the release of Bcl-2, resulting in the suppression of BECN1 and subsequent inhibition of autophagy. ER, estrogen receptor; PI3K, phosphatidyllnositol 3-kinase; AKT, protein kinase B; mTOR, mechanistic target of rapamycin; NFkB, nuclear factor kappa-light-chain-enhancer of activated B cells; PTEN, phosphatase and tensin homolog; DRAM2, DNA damage-regulated autophagy modulator-2; Bcl-2, B-cell lymphoma 2; BCN1, Beclin 1.

A growing body of evidence suggests that the key mechanism in the sex-specific regulation of autophagy is mediated by estrogens and ERs. Indeed, as suggested by bioinformatics analyses, many autophagy genes would be transcriptionally regulated by ERα and ERβ [98]. There are 12 autophagy genes regulated by ERβ, including ULK2, ATG7, ATG13, ATG14, ATG16L1, UVRAG and AMBRA1, and 19 regulated by ERα, such as ULK2, ATG5, LC3B, PIK3C3 and SQSTM1 (Table 1 and Figure 4).

However, the mechanisms by which this regulation occurs are not yet clear, as it is highly dependent on the physiological or pathological context in which it occurs. To understand how hormones can modulate autophagy, experimental confirmation in appropriate preclinical in vitro and in vivo models is necessary. Accumulating data show that ERα is involved in the transcriptional regulation of several core autophagy genes that act at stages of phagophore induction, expansion and maturation in humans. In particular, ERα has been shown to regulate, in human breast cancer, phagophore induction through the control of a large number of autophagy genes in the ULK complex and PtdIns3-K complex 1 at the transcriptional level [82,99].

Tatti and co-workers [100] have shown that ERβ plays a role in the transcriptional regulation of cathepsin D and B, lysosomal proteinases involved in autolysosomal degradation, with two estrogen-responsive elements in the promoter of the corresponding human genes.

Recent evidence has shown that there is a link between autophagy and estrogen in gastric cancer (GC). ERβ has been shown to be more abundant in GC cells than in normal gastric tissue, suggesting its pro-tumor role. Indeed, the downregulation of ERβ expression by siRNA was able to induce apoptosis and trigger autophagy in GC cells. The authors also demonstrated that pharmacologically inhibiting autophagy also suppressed apoptosis, thus suggesting that autophagy may be a prerequisite for inducing apoptosis, at least in GC cells [101].

Interestingly, an opposite role of ERβ has been demonstrated in Hodgkin lymphoma (HL). ERβ activation by agonists was indeed able to exert an antiproliferative effect, both in vitro, in a panel of HL cells, and in vivo, in non-obese diabetic/severe combined immunodeficient (NOD/SCID) mice engrafted with HL cells, by triggering the DNA damage-regulated autophagy modulator 2 (DRAM2)-dependent autophagic cascade [102].

Below, we briefly describe some examples of solid tumors, such as colorectal cancer (CRC), gastric cancer (GC), non-small cell lung carcinoma (NSCLC) and glioblastoma (GBM), and some hematological malignancies (HMs), in which estrogens and ERs play an important role in the observed sex differences at the epidemiological, clinical and/or biological level.

Table 2 shows the incidence and mortality in Europe, updated to 2022, expressed as absolute numbers, and the male/female (M/F) ratio of the above-mentioned tumors [https://gco.iarc.who.int/today (accessed on on 16 January 2025)].

### 6.1. Colorectal Cancer (CRC)

Several digestive tract tumors are characterized by a gender disparity in incidence and mortality rates that are lower in women than in men. This observation suggests a potential protective role of female steroid hormones, particularly estrogen, in the development of these tumors [103].

As far as CRC is concerned, both sex and gender differences have been observed in incidence, survival, anatomical location and molecular characteristics. Men have a higher incidence than women, and women aged 18 to 44 years have a higher survival rate than both men of the same age and older women (over 50 years, postmenopausal), suggesting that sex steroid levels may play a key role in the development of CRC [104].

It has been observed that women have a higher risk of developing CRC in the proximal intestine (right) and men in the distal intestine (left) and rectum [105]. These two different anatomical locations (left and right) actually correspond to two different diseases from a molecular point of view, with microsatellite instability (MSI) strongly characterizing left-sided CRC. A case–control study of 1836 CRC cases and 2410 population-based controls found that women were less likely than men to have MSI-positive tumors at a young age. This likelihood increased significantly with advancing age from menopause onwards. Furthermore, oral contraceptive use was associated with a lower risk of MSI+ tumors, thus suggesting a role for estrogens in protecting women from developing MSI-positive CRC. Interestingly, this study also found that obesity and a lack of physical activity were associated with an elevated risk of both MSI-positive and -negative CRC in males and females, indicating that these lifestyle factors (i.e., associated with gender rather than sex) impact men and women equally [106]. The dysregulation of serum sex steroid levels has also been observed in patients with CRC compared to healthy women. However, this pattern is not seen in men [107]. In both normal and neoplastic colon tissue, ERβ expression is predominant over ERα, but the expression of both receptors tends to decrease during tumor progression, and this reduction is associated with reduced survival in both men and women [108]. The protective role of estrogens against CRC could, therefore, pass through the activation of ERβ [109]. Scientific data from ERβ-deficient animals has shown that the absence of ERβ increases the number and size of colon tumors [110]. Conversely, increased ERα expression was associated with poor prognosis, with decreased overall survival, tumor differentiation and invasion [111,112]. Men have been observed to have increased levels of ERα [113]. It is plausible that the predominance of ERα- or ERβ-associated signaling is related more to the ratio of expression of the two receptors than to their absolute expression; an increased expression of ERβ could favor the heterodimerization of ERα–Erβ, suppressing the pro-tumor signaling triggered by ERα [114].

A protective role for ERβ has also been suggested by the observation that exposure to bisphenol A (BPA), an endocrine disruptor used to produce polycarbonate, epoxy resins, flame retardants, etc., could promote intestinal carcinogenesis because of its antagonistic effect on ERβ [115]. BPA is also reported to induce autophagy by regulating the PTEN/PI3K/AKT/mTOR pathway in pancreatic cells [116]. Based on the above, the use of some phytoestrogens with ERβ agonist activity as chemopreventive agents in patients at risk of colorectal cancer has been hypothesized [115].

### 6.2. Gastric Cancer (GC)

GC is approximately twice as common in males as in females of childbearing age, while the incidence among postmenopausal women is very similar to that found in males [117]. Furthermore, hormone replacement therapy appears to have some protective effect on the risk of developing GC, suggesting an important role for estrogens [118].

Both ERα and ERβ are present at the cellular and tissue levels in GC, and no significant difference has been observed between men and women in their expression levels. Conflicting results have been reported regarding the clinical significance of ER expression in GC. In fact, ERα expression has been associated with worse overall survival [119], but it has also been observed that high ERα expression suppressed both human GC cell proliferation and β-catenin expression in vitro [120]. Other studies have shown that although GC cells express both ERα and ERβ genes, the expression of ERβ is significantly higher than that of ERα [119,121], although ERβ expression was significantly lower in advanced GC than in early GC [122]. It should be noted that ERβ positivity in GC was associated with better 3-year survival, while its absence correlated with poor overall survival [119]. Studies regarding the role of the ERα variant ERα-36 in promoting GC indicated that its expression, both in terms of protein and mRNA, is higher in gastric tumor tissues than in normal ones, and its presence has been associated with metastasis phenomena, so much so that it can be considered an indicator of the presence of metastases [123]. However, the molecular mechanisms through which ERα-36 would exert its tumor-promoting action remain to be clarified.

### 6.3. Non-Small Cell Lung Carcinoma (NSCLC)

Lung cancer (LC), particularly NSCLC, which accounts for about 85% of lung tumors, is often diagnosed at an advanced stage, resulting in a poor prognosis, and the 5-year survival rate is less than 20% [124]. It is particularly interesting to note that while the incidence of NSCLC in men has been slightly but steadily decreasing in recent decades, it has increased in women, particularly in women of childbearing age. The latter often present with a more advanced stage, a less differentiated tumor and a greater number of metastases than men or postmenopausal women, and young non-smoking women are about two-and-a-half times more likely than men of the same age to develop lung cancer. Furthermore, it has been observed that women respond better to chemotherapy and men to immunotherapy, and survival appears to be significantly better in women at the same stage of the disease. It has, therefore, been hypothesized that female sex hormones, particularly estrogens, may represent a cause, or a contributory cause, of the above [125].

Preclinical studies showed that estrogens, especially 17β-estradiol (E2), have a strong impact on the proliferation of lung cancer cells and lung fibroblasts, both in vitro and in animal models [126,127]. NSCLC is recognized as an ER-positive tumor that expresses both ERα and ERβ, although ERβ appears to be predominant [128]. Interestingly, a recent in vitro study has highlighted how the protein encoded by the fragile-site associated tumor suppressor (FATS), a novel gene involved in cancer, was able to reduce the proliferation of NSCLC cells through the modulation of autophagic processes by physically interacting with ERβ [129].

The expression profile of ERs and their intracellular distribution in NSCLC appears quite contradictory. A large analysis of human NSCLC cell lines showed that the expression of ERβ was lower than that of ERα, with the extranuclear variant ERα-36 predominating, while ERα-66 was minimally expressed [130]. Furthermore, it has also been observed that for ERα present on the plasma membrane to activate the ERK1/ERK2 signaling pathway, thus promoting proliferation, its localization within lipid rafts mediated by palmitoylation is mandatory. In fact, treatment with a palmitoyltransferase inhibitor significantly reduced E2-induced cell proliferation in NSCLC cells [131].

A recent systematic review by Castellanos et al. [132], considering 4874 cases of NSCLC, found that there was no statistically significant difference in ERβ expression between males and females. In contrast, ERα expression varied by sex, with males having a significantly higher positivity rate than females. Furthermore, ERβ was predominantly localized in the nucleus and ERα in the extranuclear space.

Regarding the correlation between ER expression and prognosis, there is also some discrepancy in the literature. A meta-analysis published in 2015 suggested that ERβ is significantly associated with good survival in patients with NSCLC [123,133], while a more recent report showed that ERβ overexpression is not associated with prognosis [124,134]. According to Castellanos et al. [132], and in line with what has been reported by other authors, the expression of ERβ would correlate with a better prognosis, while for ERα, a correlation with a worse prognosis or no correlation was observed [135,136]. On the other hand, Kawai et al. [137] had previously reported that in patients with NSCLC, cytosolic ERα expression was associated with higher tumor grade and worse overall survival (OS), while nuclear ERβ expression, although not related to grade, was associated with significantly better OS.

It is important to consider that estrogen activity not only has direct effects on lung cancer cells but may also influence immune and stromal cells within the tumor microenvironment (TME), contributing to the establishment of a pro-tumor environment. Preclinical and clinical data show that antagonists of estrogen signaling pathways are beneficial for the treatment of NSCLC, presumably by exerting their effects not only on tumor cells but also on the cellular components of the TME.

Although quite contradictory, the emerging body of data on ERs suggests that estrogen signaling could represent a potential drug target in the treatment of NSCLC.

### 6.4. Glioblastoma (GBM)

GBM is the most common type of primary malignant brain tumor, representing approximately 60% of all brain tumors in adults. Characterized by an exceptional aggressiveness, leading to poor patient survival (median overall survival of 1.2 years), GBM shows a sex difference in incidence, being more common in males than in females (male:female ratio of 1.6) [138]. In addition, in females with GBM, a better survival rate has been observed [139]. A meta-analysis conducted by Ostrom et al. [140] showed molecular differences between the sexes in the hereditary risk of malignant glioma. In particular, a key role was attributed to EGFR in men with GBM, while TERT seems to be involved mainly in the female sex.

A sex disparity also exists in tumor anatomical location. One population-based study reported that females have a higher incidence of GBM in the right temporal lobe, while males have a higher incidence in the frontal lobe, followed by the left temporal lobe [141]. GBM cells express both ERα and ERβ. Estrogen plays a key role in the growth and repair of nerve cells by stimulating the synthesis of nerve growth factors, which have a major impact on mood, cognition and emotion. These abilities are often disrupted after the menopause due to a decrease in the levels of estrogen. The role of ERs in glioma suppression and progression remains unclear, although scientific reports suggest that ERα and ERβ have different effects. In general, ERα has been described as promoting tumorigenic pathways, while ERβ could play a tumor-suppressive role. Accordingly, the activation of ERα leads to the upregulation of the PI3K/AKT/mTOR and MAPK/ERK pathways, which are involved in autophagy, cell proliferation, survival and migration [142]. ERβ activation, on the contrary, inhibits GBM cell proliferation by inducing cell cycle arrest and promoting apoptosis [143].

For a tumor such as GBM, whose poor prognosis is due to local infiltration rather than invasion and metastases to other organs and tissues, angiogenesis plays a crucial role. The activation of ERα has been shown to increase the expression of the pro-angiogenic factor vascular endothelial growth factor (VEGF), facilitating the formation of new blood vessels and the supply of nutrients to the growing tumor. Although wild-type ERα expression is very low in adult neural stem cells, its isoform ERα-36 is expressed in glioblastoma and has been observed to correlate with tamoxifen resistance. In agreement with this, the ERα modulator SNG 162 was observed to downregulate ERα-36 expression in the nucleus while inhibiting the migration and invasiveness of the cultured human glioblastoma cell U87 [144]. Therefore, ERα-36 may represent a novel therapeutic target for GBM. Conversely, ERβ activation was reported to inhibit angiogenesis by down-regulating the pro-angiogenic signals, thereby limiting tumor growth [145].

Estrogen metabolism also has a significant impact on GBM by influencing tumor growth, progression and the response to treatment through an autocrine/paracrine mechanism. Estrogens can be synthesized in the brain from androgens by aromatase, a member of the cytochrome P450 superfamily. Aromatase has been detected in GBM tissue, suggesting that local estrogen production may contribute to tumor development and growth. Elevated aromatase levels and increased estrogen production are typically associated with a poor prognosis in GBM patients. In particular, Lin et al. [146] reported that E2 attenuated temozolomide (TMZ)-induced oxidative stress by increasing the expression of antioxidant enzymes, thereby inhibiting tumor cell death. However, contrary to the above, Honikl et al. [147] observed that the overexpression of aromatase and ERα increases survival in patients with GBM regardless of gender. Indeed, the authors showed that the combination of E2 and TMZ treatment on GBM cells reduced the viability of tumor cells and increased their sensitivity to TMZ.

It has been suggested that the long-term use of steroid hormone replacement therapy represents a predisposing factor in the oncogenesis of multifocal glioblastoma. In the same vein, it has been hypothesized that the cross-sex hormone treatment that transgender women are subjected to for many years may increase the risk of developing multiple glioblastomas due to increased levels of estrogen stimulating the growth of tumor cells [148]. However, further research is needed to support this hypothesis.

### 6.5. Hematological Malignancies (HMs)

Hematological malignancies (HMs) are the fifth most common group of cancers in economically developed regions of the world, accounting for 7% of new cancer cases worldwide [149]. HMs include lymphomas (HL, Hodgkin, and NHL, non-Hodgkin), leukemias (L) and multiple myeloma (MM).

HMs are classified as L if the cancer is initially detected in the blood, lymphomas (HL and NHL) if the primary site is in the lymph nodes or myelomas (MMs) if they are primarily located in the bones [150]. In acute leukemias, some environmental factors (biological, chemical and physical) have been observed to be associated with an increased incidence; for example, exposure to chemicals such as some organic solvents and benzene and to ionizing radiations, as well as to some viral infections (e.g., Epstein–Barr virus, herpes virus, human immunodeficiency virus, severe acute respiratory syndrome COVID-19 and human T-lymphotropic virus) [151].

All HMs arise from dysfunctional hematopoiesis, a continuous and regulated process that requires both self-renewal and the differentiation of hematopoietic stem cells (HSCs). These dysfunctions can involve hematopoietic cells of any lineage at any stage of differentiation. HSCs have high levels of ERα but little or no expression of ERβ, progesterone receptors or androgen receptors. Therefore, estrogens exert significant effects on physiological hematopoietic processes, resulting in sexual dimorphism that also influences the development of HMs [152]. In fact, males are at higher risk and have a worse prognosis for most of these neoplasms than females, thus indicating a protective role for estrogens in HMs [153]. It is important to emphasize that, in addition to estrogens, mutations in the genes present in the area of the X chromosome that escape inactivation also contribute to the sexual dimorphism observed in HMs [153]. Thus, male sex could be considered a negative prognostic factor both in the development and progression of HMs.

ERs are also expressed in all peripheral blood cells (e.g., mononuclear cells, T and B lymphocytes, NK cells, etc.), in which, unlike in HSCs, high levels of ERβ expression were found and appear to play an antitumor role. It has in fact been observed that ERβ activation by selective agonists such as silibinin was able to inhibit the proliferation of NHL human cells both in vitro and in vivo through the activation of autophagic processes [154]. Similar results were obtained by Yakimchuk et al. [155] in a mouse model of T-cell lymphoma, using both wild-type and ERβ^−/−^ animals, where ERβ agonists were able to prevent angiogenesis and tumor spread.

## 7. ERs and Autophagy as Potential Targets in Antitumor Therapy

Given the dual role of autophagy in tumor survival and drug resistance, both inhibitors and inducers of autophagy have been developed and studied in preclinical and clinical studies.

Tyrosine kinase inhibitors such as erlotinib promote autophagy in NSCLC cells, leading to cell death by excess autophagy (type 2-programmed cell death) [156]. Similarly, rapamycin, an mTOR inhibitor, triggers autophagy activation and induces cell death in several types of cancer [157]. In addition, gossypol, a BH3 mimetic, disrupts the interaction between Bcl-2 and Beclin 1 and induces autophagy and apoptosis [158,159]. In contrast, ATG inhibitors (e.g., NSC185058, which inhibits ATG4) suppress autophagosome formation, slowing tumor growth in osteosarcoma in vivo models [160]. ATG4B inhibitors with a benzotropolone core structure block autophagy and increase chemotherapy efficiency in mice [161,162]. UAMC-2526 and tioconazole interfere with the elongation and closure of the autophagosome, thereby enhancing the antitumor effects of chemotherapy [161]. Furthermore, approved drugs, such as chloroquine, hydroxychloroquine, mefloquine and bafilomycin A, prevent the fusion of lysosomes with autophagosomes, thereby inhibiting autophagy and sensitizing tumor cells to chemotherapeutic agents while promoting apoptosis [163]. The modulation of autophagy can be strategically combined with chemotherapy to more effectively disrupt tumor cell homeostasis. For example, the combination of chloroquine (CQ) and pirarubicin showed enhanced antitumor efficacy in a cervical cancer xenograft mouse model and is currently being evaluated in clinical trials [164].

In GBM, we observed that chlorpromazine (CPZ), a phenothiazine derivative used in psychiatry for more than 60 years, induced autophagy in cultured glioblastoma cells and in patient neurospheres. It synergized with TMZ in reducing GBM cell viability, while both drugs worked together to reduce cloning efficiency and induce cell death [165]. On this basis, we initiated a phase 2 clinical trial (EudraCT # 2019-001988-75; ClinicalTrials.gov identifier: NCT04224441), adding CPZ to standard GBM treatment (TMZ + radiation) in patients with a tumor with a hypomethylated MGMT gene promoter, which is characterized by resistance to TMZ and a worse prognosis. This study highlighted that the addition of CPZ to standard TMZ in the first-line treatment of patients with GBM with unmethylated MGMT gene promoters was safe and resulted in longer progression-free survival than expected in this patient population.

Furthermore, autophagy-associated antigen presentation and subsequent immune cell activation offer new opportunities for cancer immunotherapy. For example, the combination of mTOR inhibitors with inhibitors of programmed cell death protein 1 (PD-1) or PD-L1 effectively reduced tumor growth in hepatocellular carcinoma [166].

Although preclinical studies have suggested a link between autophagy and ERs in resistance phenomena, no clinical studies have been conducted combining autophagy modulators (both inhibitors and activators) with ER ligands.

In glioblastoma, it has been observed that some selective ER modulators, including raloxifene, were able to promote tumor cell death by inducing a blockade of autophagic processes [167].

Furthermore, the prenylated flavonoid icaritin, currently undergoing phase 3 clinical trials for the treatment of advanced hepatocellular carcinoma, has been shown to be able to modulate the splice variant of ERα, ERα-36, and by acting on the apoptosis/autophagy crosstalk, to carry out significant antitumor activity [168].

In esophageal carcinoma cells, it has been observed that the activation of the ERβ–ROS pathway inhibited autophagy by enhancing DNA damage, thus sensitizing chemo-resistant tumor cells to fluorouracil (5FU)-induced cell death.

As far as immunotherapy is concerned, it has been recently observed that the E2/ERα ratio was able to predict the response to pembrolizumab in patients with NSCLC, also suggesting the use of aromatase inhibitors as possible sex-specific immunoadjuvants in this type of tumor [169].

While a better response has been observed in women to traditional chemotherapy [170], a sex disparity in favor of men has been reported in immunotherapy, in which estrogens probably play an important role [171]. However, although immune checkpoint inhibitors have emerged as a standard of care for the treatment of many different types of cancer, data on the different efficacy of immunotherapy between men and women are still contradictory and not definitive. The observed discrepancies have, however, been hypothesized to depend on sex hormones, in particular estrogens, which play an important role in the functioning of the immune system in physiological conditions [172].

Even in terms of the adverse effects of both chemotherapy and immunotherapy, published data are contradictory, with a prevalence of studies that would seem to indicate that side effects are lower in males than in females [173].

It is, therefore, a scientific priority to investigate the role played by sex hormones in the efficacy and toxicity of traditional, targeted and immunological antineoplastic therapies. Indeed, in the era of the personalized approach to therapy, the impact of the sex/gender of the people treated cannot be ignored.

## 8. Conclusions

In this review, we explore the critical role that autophagy and estrogen receptors play in cancer development and progression and in determining sex differences.

Autophagy is a fundamental cellular process involved in the degradation and recycling of cellular components. It is highly regulated by signaling pathways that allow cells to adapt to environmental and metabolic changes. It plays a dual and complex role in cancer, acting as both a tumor suppressor and a tumor promoter, depending on the stage and context of tumor development. In early cancer, it suppresses tumorigenesis by maintaining genomic stability, limiting inflammation and reducing oxidative stress. In contrast, in advanced cancers, autophagy promotes survival by facilitating metabolic adaptation, ensuring mitochondrial homeostasis and contributing to immune evasion and drug resistance. This duality highlights the need for a diversified approach in the development of autophagy-based cancer therapies. In particular, autophagy intersects with sex-specific biological differences, as sex hormones and genetic factors play an important role in its modulation. Estrogen receptors, particularly ERα and ERβ, further contribute to gender disparities by exerting effects at multiple levels: in autophagy, tumor development and immunological response. While ERβ often acts as a tumor suppressor by promoting autophagic clearance and apoptosis, ERα is more frequently associated with pro-tumor signaling pathways.

All these aspects contribute in a complex way to the observed sex differences in the incidence, progression and outcomes of a large number of cancers, including colorectal cancer, gastric cancer, lung cancer, glioblastoma and the hematological malignancies mentioned in this work.

Autophagy significantly influences the response to cancer therapies, including chemotherapy, targeted therapy and immunotherapy. The complex and non-unique involvement of autophagy in tumors is evidenced by numerous clinical and preclinical studies that have shown that both autophagy inducers, such as tyrosine kinase inhibitors and rapamycin, and its inhibitors, such as chloroquine and specific ATG protein blockers, can improve the efficacy of cancer treatments by opposing tumor survival mechanisms. Targeting autophagy sensitizes tumor cells to therapeutic agents by compromising their ability to resist metabolic stress and immune attack. The combination of autophagy modulators with radiotherapy, traditional chemotherapeutics, molecularly targeted drugs or immune checkpoint inhibitors therefore represents a promising therapeutic strategy.

The mutual interaction between autophagy and estrogens, through ERs, offers new opportunities for sex-specific personalized cancer therapy. Indeed, estrogens affect autophagic processes, suggesting potential therapeutic benefits of combining ER modulators with autophagy regulators.

However, contradictory results regarding the role of ERs in tumors suggest caution and further investigations to identify effective sex-specific treatment strategies.

The sex disparity observed in tumors, also in terms of treatment response and toxicity, highlight the importance of incorporating sex and gender in the design of epidemiological and experimental studies, both in vitro and on animal models, as well as the enrollment of a significant number of women in clinical trials.

In conclusion, significant differences in genetics, anatomy and physiology between women and men determine sex differences in tumor onset and progression as well as in the efficacy and toxicity of therapy. Many factors are responsible for these differences; among them, sex hormones that can regulate autophagic processes, the alteration of which is often found in cancer and is associated with resistance to therapy. So far, the molecular mechanisms underlying sex differences in autophagy have not been well understood. Understanding how estrogen/autophagy crosstalk may contribute to the observed sex disparities in cancer represents an opportunity to identify new molecular targets, overcome resistance and improve survival in patients with cancer.

## Figures and Tables

**Table 1:** Autophagy genes regulated by ERs.

Phases Autophagy	ERα	ERβ	ERα/β
Phagophore Induction	ATG2A, PI3KC, ATG9A	UVRAG	Ambra1, ULK2, ATG14, ATG13
Phagophore Expansion	ATG5, LC3B, SQSTM1, ATG4C, ATG4D, ATG7	ATG10, ATG16L1	ATG4B, ATG10, ATG16L2, ATG7
Fusion			TECPR1, FYCO1

**Table 2:** Cancer incidence and mortality in Europe (updated to 2022).

	Colorectal Cancer	Gastric Cancer	Lung Cancer	Glioblastoma	Hematological Malignancies (NHL, HL, L, MM)
Incidence absolute numbers	538,262	135,610	484,306	67,559	306,921
Sex disparity in incidence M/F ratio	1.3	1.4	2.0	1.2	1.2 *
Mortality absolute numbers	247,842	95,431	375,569	54,001	228,961
Sex disparity in mortality M/F ratio	1.2	1.4	2.0	1.2	1.2

* Follicular lymphoma represents an exception (M/F = 0.9).

## Data Availability

Not applicable.
